# The Role of Multiple Neuromodulators in Reinforcement Learning That Is Based on Competition between Eligibility Traces

**DOI:** 10.3389/fnsyn.2016.00037

**Published:** 2016-12-15

**Authors:** Marco A. Huertas, Sarah E. Schwettmann, Harel Z. Shouval

**Affiliations:** ^1^Department of Neurobiology and Anatomy, University of Texas Medical SchoolHouston, TX, USA; ^2^Department of Computational and Applied Mathematics, Rice UniversityHouston, TX, USA; ^3^Department of Brain and Cognitive Sciences, Massachusetts Institute of TechnologyCambridge, MA, USA

**Keywords:** reinforcement-learning, synaptic plasticity, eligibility-trace, LTP, LTD, reward, neuromodulator, timing

## Abstract

The ability to maximize reward and avoid punishment is essential for animal survival. Reinforcement learning (RL) refers to the algorithms used by biological or artificial systems to learn how to maximize reward or avoid negative outcomes based on past experiences. While RL is also important in machine learning, the types of mechanistic constraints encountered by biological machinery might be different than those for artificial systems. Two major problems encountered by RL are how to relate a stimulus with a reinforcing signal that is delayed in time (temporal credit assignment), and how to stop learning once the target behaviors are attained (stopping rule). To address the first problem synaptic eligibility traces were introduced, bridging the temporal gap between a stimulus and its reward. Although, these were mere theoretical constructs, recent experiments have provided evidence of their existence. These experiments also reveal that the presence of specific neuromodulators converts the traces into changes in synaptic efficacy. A mechanistic implementation of the stopping rule usually assumes the inhibition of the reward nucleus; however, recent experimental results have shown that learning terminates at the appropriate network state even in setups where the reward nucleus cannot be inhibited. In an effort to describe a learning rule that solves the temporal credit assignment problem and implements a biologically plausible stopping rule, we proposed a model based on two separate synaptic eligibility traces, one for long-term potentiation (LTP) and one for long-term depression (LTD), each obeying different dynamics and having different effective magnitudes. The model has been shown to successfully generate stable learning in recurrent networks. Although, the model assumes the presence of a single neuromodulator, evidence indicates that there are different neuromodulators for expressing the different traces. What could be the role of different neuromodulators for expressing the LTP and LTD traces? Here we expand on our previous model to include several neuromodulators, and illustrate through various examples how different these contribute to learning reward-timing within a wide set of training paradigms and propose further roles that multiple neuromodulators can play in encoding additional information of the rewarding signal.

## 1. Introduction

In order to maximize reward, animals must be able to relate predictive stimuli to future reward and estimate the expected timing and magnitude of the reward. Although, animals can behaviorally accomplish all of these aims, the physiological basis for this behavior is not yet evident. In any case, any general mechanism for learning associations between stimulus and reinforcement have to overcome two challenges. First, learning usually occurs when reward is delayed with respect to stimulus offset. This poses the problem of how the association between a stimulus and a delayed reward is established; this is often called the temporal credit assignment problem. The second challenge is that once the correct association is established, learning should stop, which calls for the implementation of a stopping rule. Many theoretical learning rules have been proposed to solve such problems (Sutton and Barto, 1998). One of the more biologically plausible ideas is that the temporal credit assignment is solved via eligibility traces. These are physiological processes that get activated by the stimulus and, in its absence, decay slowly, thus bridging the temporal gap between the stimulus and a delayed reward. Eligibility traces are converted to actual changes in network function if a reward arrives while they are still active. Once the network learns to predict the properties of the expected reward, the goal of learning has been attained and further changes to the network should stop. Therefore, when the predicted reward matches the actual reward (i.e., no reward prediction error), plasticity should be inhibited. Mechanistically, this can be implemented if the networks internal prediction of reward inhibits the reward nucleus so that no further reward signal is provided.

At the physiological level, cells have been shown to change their response to stimuli as a result of reinforcement learning paradigms (Beitel et al., 2003; Yin et al., 2014). Recent experimental evidence also shows that brain cells can indeed learn to associate stimuli with delayed reward, and that the temporal dynamics of such cells can predict expected reward-times (Shuler and Bear, 2006; Chubykin et al., 2013). The cellular and network mechanisms that allow the representation and learning of these associations and their related reward times are not yet clear. Models of recurrent networks with RL based plasticity rules can learn to emulate the dynamics of these cortical neurons (Gavornik et al., 2009; Gavornik and Shouval, 2010). The RL rule used in these network models includes both synaptic eligibility traces and the inhibition of the reward signal/nucleus. In such networks, inhibition of a reward that is delayed with respect to the stimulus is possible because the network learns to be active up until the expected reward time. Therefore, such simulated networks can learn when to expect a reward, and are able to inhibit the reward in order to stabilize learning.

Experiments in visual cortex slices as well as *in vivo* have identified the neuromodulator that allows these circuits to learn to predict reward timing, and unlike in many other systems, this neuromodulator is not dopamine but acetylcholine (Chubykin et al., 2013). Further, learning stable physiological representations can be mimicked by directly delivering the neuromodulator, either pharmacologically in slice (Chubykin et al., 2013) or with optogenetic stimulation *in vivo* (Liu et al., 2015). In these experiments, inhibition of reward is not possible since the putative negative feedback loop was eliminated from the experimental model, yet stable learning is achieved. Therefore, these experiments cast doubt on the biological validity of a stopping rule based on reward inhibition, at least in some brain systems. We therefore set out to design a biophysically plausible learning rule in which explicit inhibition of reward is not needed.

To account for these findings, we recently described a model of competitive reinforcement learning (CRL) in which the stopping rule is implemented by competition between two different eligibility traces: one for long-term potentiation (LTP) and the other for long-term depression (LTD) (He et al., 2015). These traces have different dynamics that depend on pre and postsynaptic cortical activation and are converted to changes in synaptic efficacy in the presence of neuromodulators. We have demonstrated theoretically that such a CRL can be used in recurrent networks to learn the expected time of reward and attain stability via a stopping rule that derives from the temporal competition between the two eligibility traces without the need to invoke the inhibition of the reward signal. Although, until recently the concept of synaptic eligibility traces had no experimental support, evidence for their existence has now been provided (Cassenaer and Laurent, 2012; Yagishita et al., 2014; He et al., 2015).

The CRL model requires a single neuromodulator to convert both the LTP and LTD traces into synaptic efficacies. Experimentally it has been found that there are at least two different neuromodulators that are used to translate eligibility traces into synaptic plasticity, and that different ones are used for LTP and LTD (He et al., 2015). For instance, in layer 2–3 of neocortex, norepinephrine is necessary for expressing LTP traces and serotonin for converting LTD traces. In prefrontal slices, norepinephrine and dopamine can convert LTP traces while serotonin converts LTD traces. The question posed in this paper is thus: what are the advantages of a system in which multiple neuromodulators are used selectively for different purposes? We will show two new examples, derived from an extended version of our original CRL model, in which the existence of different neuromodulators can play a significant role. In one example we show that the implementation of a novel ramp-reward learning paradigm (Namboodiri et al., 2015) in a recurrent network is aided by two different neuromodulators. In another example we show that a feed-forward network employing two different neuromodulators is able to learn about the reward magnitude. In general these results demonstrate that multiple neuromodulators can make the system more flexible and stable.

## 2. Results

### 2.1. A model of stable reinforcement learning based on competition between eligibility traces

The CRL is based on the assumption that at every synapse, two synaptic eligibility traces, one for LTP and one for LTD, can simultaneously be activated through the temporal firing patterns of the pre- and post-synaptic cells in a Hebbian manner. Moreover, the activity of these traces can saturate at different levels and in the absence of any cellular activity their activation level decays with different time constants. These assumptions of the temporal dynamics of these traces can be formulated mathematically as a pair of coupled differential equations of the form:

(1) $$\begin{array}{r} \tau_{p}\frac{dT_{ij}^{p}}{dt}=-T_{ij}^{p}+H^{p}\left(S_{i},S_{j}\right)\frac{\left(T_{max}^{p}-T_{ij}^{p}\right)}{T_{max}^{p}} \end{array}$$

(2) $$\begin{array}{r} \tau_{d}\frac{dT_{ij}^{d}}{dt}=-T_{ij}^{d}+H^{d}\left(S_{i},S_{j}\right)\frac{\left(T_{max}^{d}-T_{ij}^{d}\right)}{T_{max}^{d}} \end{array}$$

where the labels *p* and *d* refer to LTP and LTD synaptic eligibility traces, respectively. Thus, for *a* ∈ {*p*, *d*}, $T_{ij}^{a}$ represents the activation of the synaptic eligibility trace at a synapse labeled by the index *ij*, (i.e., between the *j*-th presynaptic and *i*-th postsynaptic cells), τ_*a*_ is the characteristic decay time constant, and $T_{\text{max}}^{a}$ sets the saturation level for the activation of the trace. *H*^*a*^ stands for the Hebbian rule, which itself depends on the firing rates or spike patterns of the pre- and post-synaptic cells *S*_*j*_(*t*) and *S*_*i*_(*t*), respectively. In general there can be different Hebbian rules for every synapse and thus we can have $H^{a}\left(S_{i},S_{j}\right)=H_{ij}^{a}$. In many applications we will use a rate-dependent Hebbian rule of the form $H_{ij}^{a}=S_{i}S_{j}$, where the *S*_*k*_, the average firing rates, are computed using Equation (25). The above equations can be cast in a more familiar form

(3) $$\begin{array}{l} \frac{\;dT^{a}}{dt}=-\frac{1}{{\tilde{\tau }}^{a}}\left(T^{a}-{\tilde{T}}^{a}\right) \end{array}$$

where the indices *i*, *j* have been dropped, ${\tilde{\tau }}^{a}$ is the effective time constant and ${\tilde{T}}^{a}$ is the effective saturation activity of the trace given by

(4) $$\begin{array}{r} {\tilde{T}}^{a}=\frac{T_{max}^{a}H^{a}}{T_{max}^{a}+H^{a}}\text{and} \end{array}$$

(5) $$\begin{array}{r} {\tilde{\tau }}^{a}=\frac{T_{max}^{a}\tau^{a}}{T_{max}^{a}+H^{a}}. \end{array}$$

The general solution of Equation (3) for a time-dependent Hebbian term, *H*^*a*^ = *H*^*a*^(*t*), is

(6) $$\begin{array}{r} T^{a}=\exp\left(-\int_{0}^{t}\frac{du}{{\tilde{\tau }}^{a}\left(u\right)}\right)\\ \left\{\int_{0}^{t}\frac{{\tilde{T}}^{a}}{{\tilde{\tau }}^{a}}\exp\left(\int_{0}^{v}\frac{du}{{\tilde{\tau }}^{a}\left(u\right)}\right)dv+T^{a}\left(0\right)\right\} \end{array}$$

(7) $$\begin{array}{rc} = & \exp\left[-\frac{1}{\tau }\int_{0}^{t}\left(1+\frac{H^{a}\left(u\right)}{T_{max}^{a}}\right)du\right]\times \\ \left\{\frac{1}{\tau }\int_{0}^{t}H^{a}\left(v\right)\exp\left[\frac{1}{\tau }\int_{0}^{v}\left(1+\frac{H^{a}\left(u\right)}{T_{max}^{a}}\right)\;du\right]dv+T^{a}\left(0\right)\right\} \end{array}$$

where *T*^*a*^(0) is the activity of the trace at time *t* = 0.

In order to gain some insight into the dynamics of the traces we consider the special case of a constant Hebbian term, i.e., *H*^*a*^ = const. Assuming that *H*^*a*^ is non-zero during a period of time (rising phase) and then turns off (falling phase), the dynamics of the traces is as follows. When *H*^*a*^ is non-zero, the trace dynamics will converge exponentially to the non-zero level.

Thus, during the rising phase (*H*^*a*^ > 0), the dynamics of the eligibility trace take the form

(8) $$\begin{array}{r} T_{R}^{a}\left(t\right)={\tilde{T}}^{a}\left(1-\exp\left(-\frac{t}{{\tilde{\tau }}^{a}}\right)\right) \end{array}$$

where *R* labels this solution for the rising phase.

**Figure 1:**
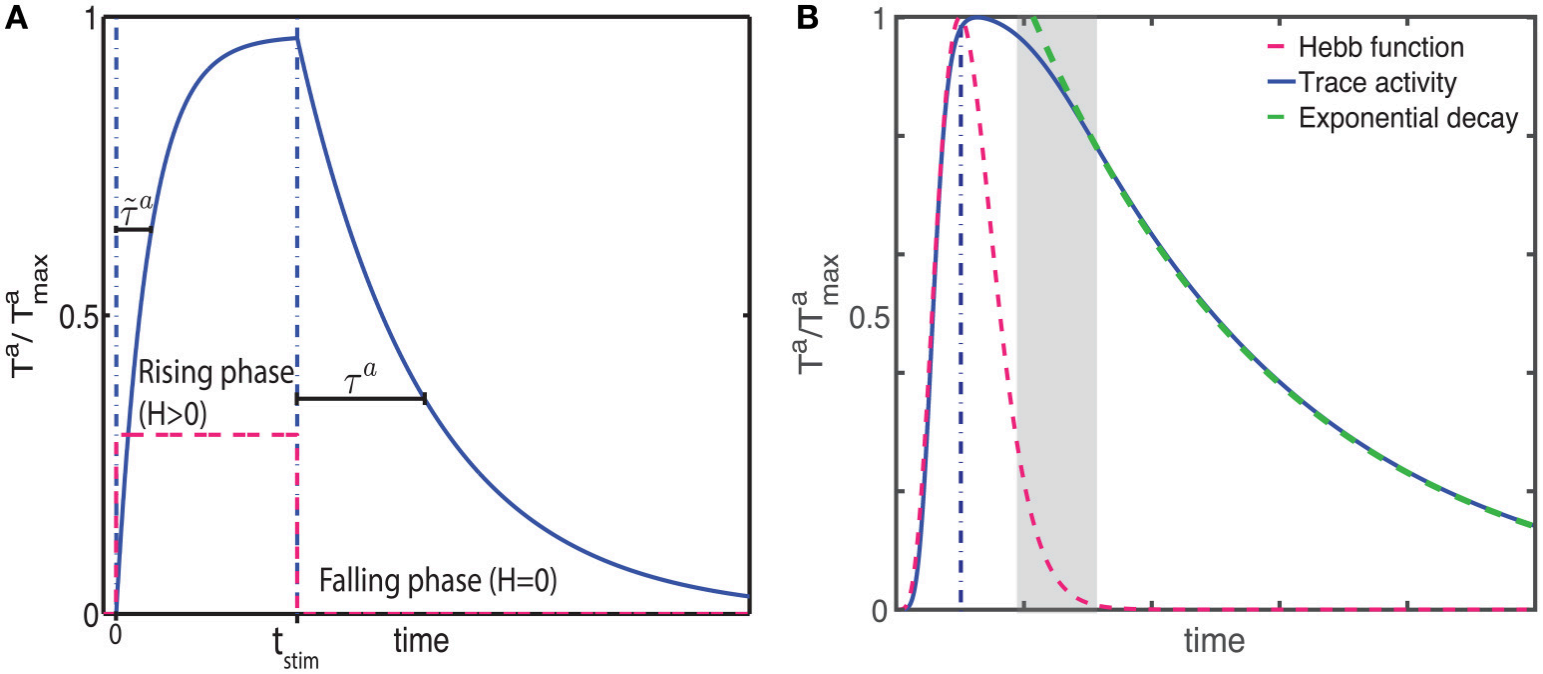
**Examples of trace activity for different time-dependent Hebbian functions**. **(A)** Temporal profile of synaptic eligibility traces for a square Hebbian stimulus, illustrated by the dashed red line, with onset at time *t* = 0 and offset at time *t* = *t*_stim_. Traces rise with a time constant ${\tilde{\tau }}^{a}$ (Equation 5) to an upper steady state ${\tilde{T}}^{a}$ (Equation 4) and decay to zero with a slower time constant τ^*a*^. **(B)** A smooth, time-varying Hebbian function (dashed red line) representing the contributions of pre- and post-synaptic cell activity and the resulting synaptic eligibility trace obtained from Equation (7) (blue). After the Hebbian term has decayed to zero, the dynamic of the trace follows an exponential decay (green).

During the falling phase (*H*^*a*^ = 0), the trace will decay to zero with a time constant τ^*a*^ following an exponential decay (Figure 1A)

(9) $$\begin{array}{l} T_{F}^{a}\left(t\right)=T_{R}^{a}\left(t_{\text{stim}}\right)\;e^{-\left(t-t_{\text{stim}}\right)/\tau^{a}} \end{array}$$

where *F* labels this solution for the falling phase and $T_{R}^{a}\left(t_{\text{stim}}\right)$ is the activity of the trace at the end of the rising phase (*t* = *t*_stim_). The time course of the trace activity for this example is illustrated in Figure 1A.

Note that these equations are based on the simplifying assumption that *H*^*a*^ is constant throughout the different phases. However, in general the Hebbian term *H*^*a*^ will vary in time and the dynamics of the traces will deviate from the simple description given earlier. Since in truth *H*^*a*^ will fluctuate over time and from trial to trial, the equations describing the activity of the traces characterize the average behavior.

**Figure 2:**
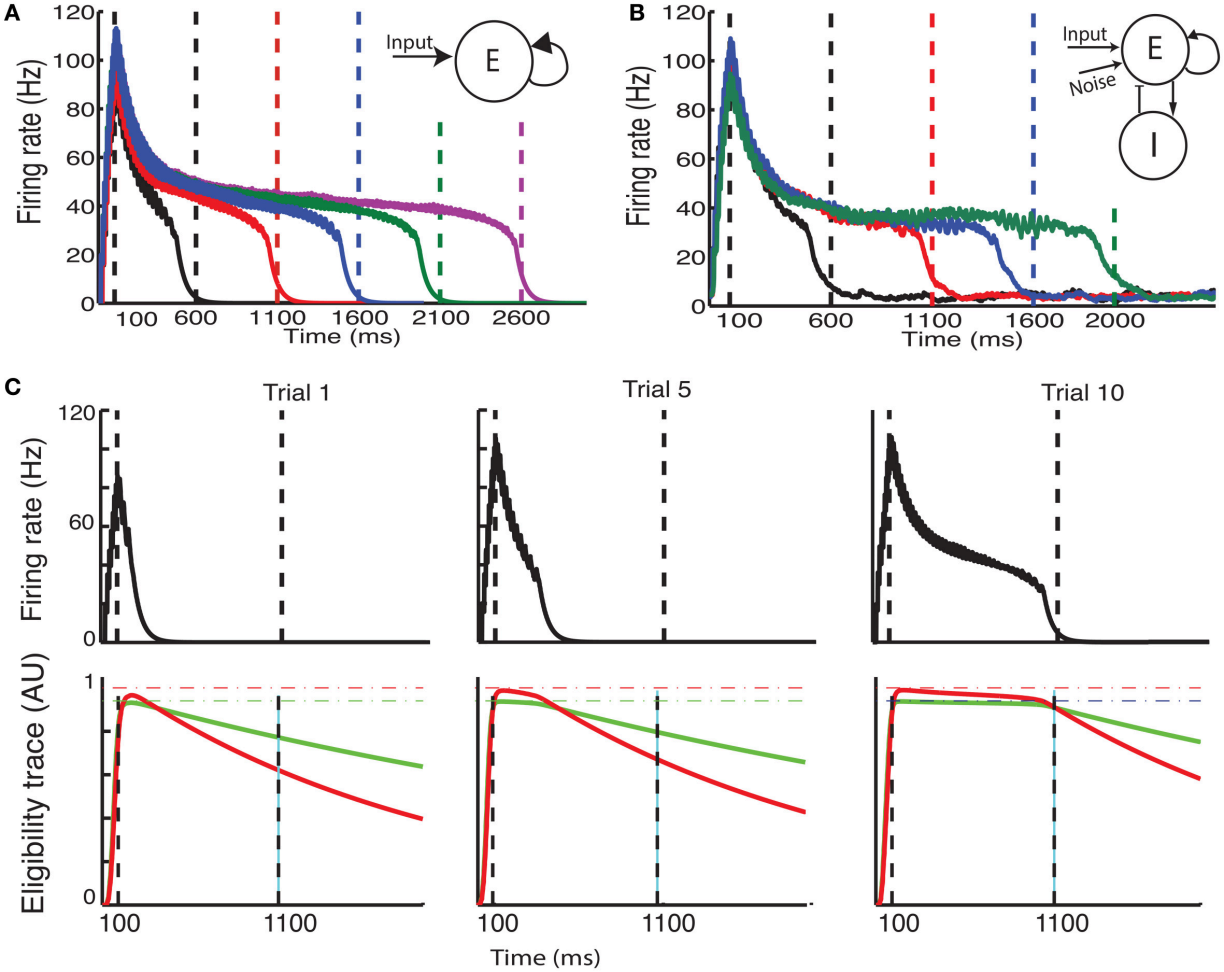
**The CRL in a recurrent neural network**. **(A)** With a two-trace rule, an integrate-and-fire network with excitatory recurrent connections can learn to generate different interval times. **(B)** In a two-population network with fixed inhibitory connections, plastic excitatory connections, and external noise, a large range of interval times can be learned as well. **(C)** Illustration of the learning process in a recurrent network. Initially (Trial 1, left) the network activity (top panel) quickly decays and so do the LTP (green) and LTD (red) associated eligibility traces (bottom panel). At the time of reward (vertical dashed line) the LTP trace dominates and recurrent connections are strengthened resulting in longer lasting network activity. After 5 trials (middle panel) the network activity lasts for longer, and traces reach saturation (dashed lines). At the time of reward LTP traces still dominate, resulting in an extension of the network time constant. After 10 trials (right), network activity extends almost to the time of reward, and the traces are equal at the time of reward. Therefore, there is no change in the strength of lateral connection and the synaptic plasticity reaches a steady state.

An example of the temporal dynamics of the activity of the trace for a smoothly time-varying Hebbian term is given in Figure 1B. In this example, the activity of the trace increases rapidly in a non-exponential way during the rising phase. As the magnitude of the Hebbian term starts decreasing, the initial decay of the trace is slow but as the magnitude of the Hebbian term decreases to zero (gray shaded area), the activity follows an exponential decay, illustrated by the green-dotted line, with its characteristic decay constant τ^*a*^. Further examples from actual simulation are given in Figure 2C.

Throughout this paper we use the simplified expression $H_{ij}^{a}=\eta_{a}H_{ij}$, to indicate that the Hebbian terms for LTP and LTD are identical up to a multiplicative constant. Under this assumption Equations (4, 5) are transformed to:

(10) $$\begin{array}{r} {\tilde{T}}_{ij}^{a}=T_{\text{max}}^{a}\frac{\eta_{a}H_{ij}}{\left(T_{\text{max}}^{a}+\eta_{a}H_{ij}\right)} \end{array}$$

with time constant

(11) $$\begin{array}{r} {\tilde{\tau }}_{ij}^{a}=\tau^{a}/\left(1+\eta_{a}H_{ij}/T_{\text{max}}^{a}\right) \end{array}$$

Given these synaptic eligibility traces, we will further assume that synaptic efficacies are only modified in the presence of time dependent reward signals, which are most likely to be neuromodulators. This assumption can be simply expressed by the equation:

(12) $$\begin{array}{r} \frac{dL_{ij}}{dt}=\eta \;[R^{p}\left(t\right)T_{ij}^{p}-R^{d}\left(t\right)T_{ij}^{d}] \end{array}$$

where *L*_*ij*_ is the synaptic efficacy between neurons *i* and *j*, and *R*^*p*^, *R*^*d*^ are the time dependent reward signals that convert the eligibility traces for LTP and LTD, respectively, to synaptic efficacy and η is the learning rate. For many properties of the learning rules a single neuromodulator is sufficient, in which case *R*^*p*^ = *R*^*d*^. The temporal profile of the neuromodulator release is assumed to be shorter than the duration of the trial, *T*_trial_.

The solution of this equation has the form:

(13) $$\begin{array}{r} L_{ij}\left(T_{\text{trial}}\right)=\eta \int_{0}^{T_{\text{trial}}}[R^{p}\left(t\right)T_{ij}^{p}-R^{d}\left(t\right)T_{ij}^{d}]\;dt+L_{ij}\left(0\right) \end{array}$$

where *L*_*ij*_(0) is the synaptic efficacy at the beginning of the trial. Steady-state is obtained when the synaptic efficacy no longer changes, i.e., *L*_*ij*_(*T*_trial_) = *L*_*ij*_(0).

We will consider several simple cases for the temporal profile of neuromodulators. The simplest model, which was used previously (Huertas et al., 2015), assumes that $R^{p}\left(t\right)=R^{d}\left(t\right)=R\delta \left(t-t_{\text{reward}}\right)$ where *t*_reward_ is the time that the reward signal arrives in the cortical area. When examining the effects of different neuromodulators, we will use a model of the form $R^{p}\left(t\right)=R_{0}^{p}\delta \left(t-t_{\text{reward}}\right)$ and $R^{d}\left(t\right)=R_{0}^{d}\delta \left(t-t_{\text{reward}}\right)$ where $R_{0}^{d}\neq R_{0}^{p}$. Another possibility considered here is that the neuromodulator level is constant within an time interval between *t*_1_ and *t*_2_. Obviously these are very simple equations, but they can be replaced with more biophysically realistic ones without a qualitative change in the behavior of these rules. In general the two neuromodulators might have different release profiles and might not only depend of reward, but possibly also on expected reward or on action.

As described previously by He et al. (2015) and as will be described in the following sections, we find that in order to obtain stable reinforcement learning, the saturation level of the LTD associated trace must be larger than the saturation of the LTP trace ($T_{\text{max}}^{d}>T_{\text{max}}^{p}$). Further, to obtain learning with a delayed reward in a recurrent network, the decay of the LTP trace must be slower than that of the LTD trace (τ_*p*_ > τ_*d*_). We also show below that to obtain stable learning in a feedforward network, the rise time of the LTP trace must be faster than the rise time of the LTD trace (${\tilde{\tau }}^{p}<{\tilde{\tau }}^{d}$).

### 2.2. CRL in a recurrent spiking neural network: prediction of reward timing

We characterize the consequences of CRL in a network of integrate-and-fire neurons, as described in the Methods section, using the parameters in Table 1. We have previously shown (He et al., 2015) that with this rule it is possible to train the network to report reward times, in slowly decaying sustained neurons in V1 (Shuler and Bear, 2006). In Figure 2 we show a summary of these results. It shows that the network is able to learn spike timing for a broad range of reward times in a noise free network composed of only excitatory neurons (Figure 2A), as shown previously (He et al., 2015). We also show similar results in a network composed of both inhibitory and excitatory neurons and with realistic levels of variability (Figure 2B).

**Table 1:** **Description and values of model parameters used in simulations**.

**Parameter**	**Value**	**Units**	**Description**
*N*	100	–	Number of neurons in populations
*dt*	0.1	ms	Time step for integration
*E*_*L*_	−60	mV	Leak current reversal potential
*g*_*L*_	10 × 10^−3^	μS	Leak current conductance
*C*	20 × *g*_*L*_	nF	Membrane Capacitance
*E*_*E*_, *E*_*I*_	−5, −70	mV	Reversal potential excitatory and inhibitory currents
ρ	1/7	–	Fractional change of synaptic activation
ν_θ_	−55.0	mV	Spiking threshold
ν_*reset*_	−61.0	mV	Reset voltage
*t*_*ref*_	2.0	ms	Absolute refractory period
$\tau_{s}^{EE}$, $\tau_{s}^{EI}$, $\tau_{s}^{IE}$	80, 10, 10	ms	Time constant for synaptic activation for excitatory to excitatory/inhibitory (EE, EI, IE) connections
$\tau_{s}^{LGN}$, $\tau_{s}^{BG}$	10, 10	ms	Time constant for synaptic activation for LGN and random background (BG) inputs
*f*_*BG*_	10	Hz	Background firing rate
τ^*p*^, τ^*d*^	5000, 1500	ms	Decay time constant LTP (P) and LTD (D) synaptic eligibility traces
$T_{max}^{p}$, $T_{max}^{d}$	1, 0.92	–	Synaptic eligibility traces saturation level
η	0.5 × 10^−6^	ms^−1^	Learning rate
*W*_*IE*_, *W*_*EI*_	19 × 10^−3^, 10 × 10^−3^	μS	Synaptic connection strength from excitatory to inhibitory (IE) and inhibitory to excitatory (EI)
*W*_*LGN*_, *W*_*BG*_	100 × 10^−3^, 30 × 10^−3^	μS	Synaptic connection strength form LGN and background
τ_*r*_	50	ms	Time window for integration of firing rate

The process of training a recurrent network with the two-trace model is shown Figure 2C. Initially (Figure 2C, top left) the network activity decays quickly to baseline after the end of the stimulus, and at the time of reward the LTP related trace (Figure 2 bottom left) is larger than the LTD trace, due to the longer time constant of the LTP trace. This results in a potentiation of the synaptic efficacies, leading to longer lasting network activity (Figure 2C top, center). During learning, at the time of reward, the LTP trace is still larger than the LTD trace (Figure 2C bottom, center), leading to further enhancement of synaptic efficacies. Finally when the network activity approaches the time of reward (Figure 2C top, right) the LTP and LTD traces become equal (Figure 2C bottom, right), the synaptic efficacies no longer change, and the state of the network is stabilized.

Due to the strong connection between neurons, and in particular in the noiseless case, the neurons responses can become synchronized. However, in the noisy case responses becomes more asynchronous, especially after the stimulus offset as the time approaches the expected reward time. The simulations shown in Figures 2A,B illustrate the ability of the network to represent different times. Longer decay times are implemented by stronger recurrent weights. However, for large enough weights the firing rates in the system no longer decay and it becomes bi-stable as it was shown in Gavornik and Shouval (2010). Although, for a deterministic system with infinitesimal precision it is possible to implement any decay time, for stochastic systems the proximity to the bi-stable regime imposes a practical upper limit to the reward time the system can represent (about 2.0–3.0 s).

The balancing of the LTP and LTD traces at the learned time is accomplished in part due to the difference in the characteristic time constants of the corresponding traces. The assumption that the LTP trace decays slower than the LTD trace i.e., τ^*p*^ > τ^*d*^ has experimental support (He et al., 2015). A second condition required is that the saturation of the LTD trace is higher than that of the LTP trace. This can be understood using the simple model illustrated in Figure 1A and the related equations. For instance, during the falling phase, the time dependence of the traces can be written as

(14) $$\begin{array}{r} T^{p}={\tilde{T}}^{p}e^{-\left(t-t_{\text{stim}}\right)/\tau^{p}} \end{array}$$

(15) $$\begin{array}{r} T^{d}={\tilde{T}}^{d}e^{-\left(t-t_{\text{stim}}\right)/\tau^{d}} \end{array}$$

where ${\tilde{T}}^{a}$ (*a* ∈ {*p*, *d*}) is the saturation of the corresponding trace at *t* = *t*_stim_ (see Equation 4). If both traces balance at *t* = *t*_*r*_ then these two equations combine to give the relationship

(16) $$\begin{array}{l} \ln\;\left(\frac{{\tilde{T}}^{d}}{{\tilde{T}}^{p}}\right)=\left(t_{r}-t_{\text{stim}}\right)\left(\frac{1}{\tau^{p}}-\frac{1}{\tau^{d}}\right). \end{array}$$

From here it follows that since τ^*p*^ > τ^*d*^ it must be true that ${\tilde{T}}^{d}>{\tilde{T}}^{p}$, moreover as illustrated in Figure 2C, traces effectively saturate near their maximally allowed values $T_{\text{max}}^{a}$, and thus it follows that $T_{\text{max}}^{d}>T_{\text{max}}^{p}$ is necessary for convergence.

### 2.3. Learning reward timing with a ramp protocol: two neuromodulators facilitate stability and flexibility

**Figure 3:**
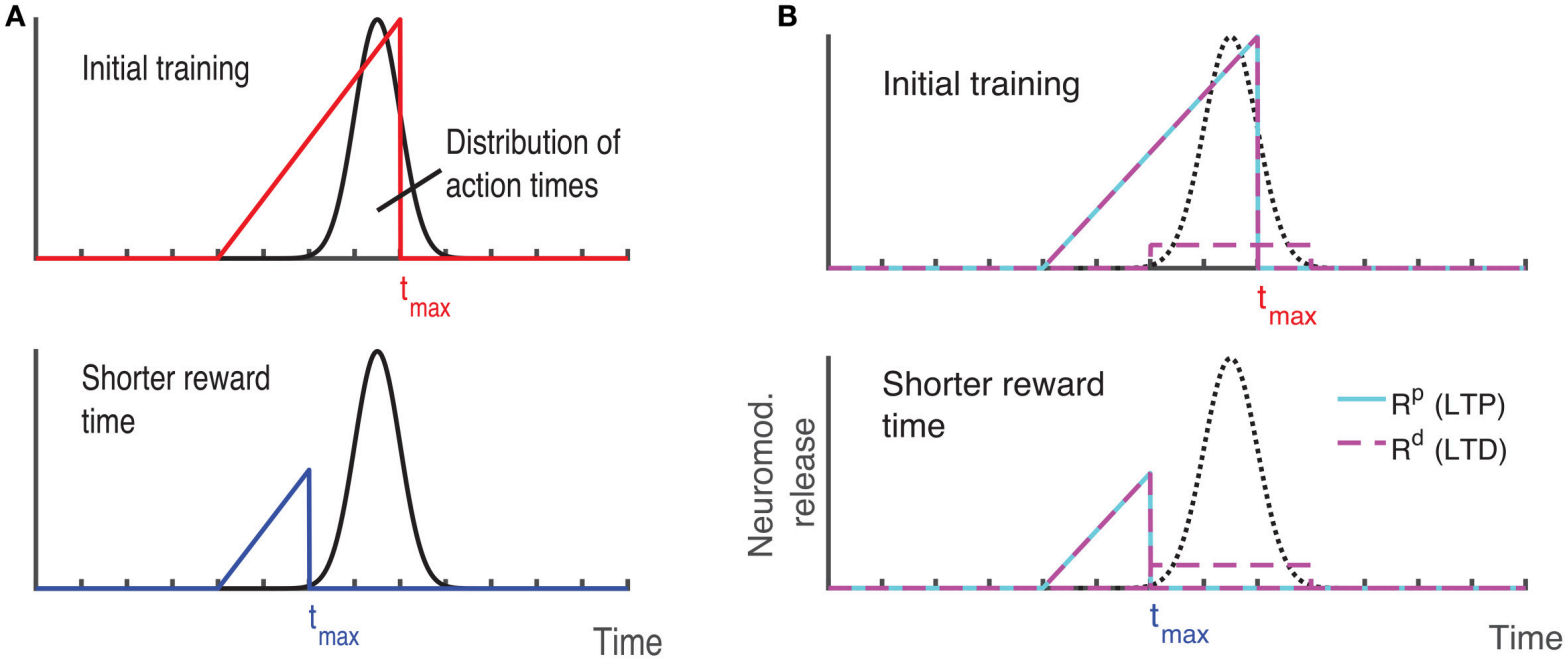
**Ramp reward paradigm and neuromodulator release profile**. **(A)** Single neuromodulator case. The reward magnitude as a function of response time is shown by the red line in the upper panel. The longer the subject waits, the bigger the reward, until a time *t*_max_ after which no reward is received. It is assumed that the magnitude of the single neuromodulator follows this profile as well. The black curve in the upper panel shows schematically the distribution of response times after learning is complete. In the lower panel a situation in which the maximum reward time (*t*_max_) is decreased (blue) while the action time responses have not yet changed in response to the new reward paradigm. Because the actions occur in the unrewarded region, the network cannot learn this new condition. **(B)** Two neuromodulator case. The reward paradigm is the same as in **(A)**, but the two neuromodulators respond differently. The two neuromodulator profiles are depicted by different colors as defined in the legend (cyan for LTP and magenta for LTD related traces). For the long reward time (upper panel), both neormodulators have the same profile if there is a reward. However, if there is an action but no reward, only the LTD related neuromodulator is released. This becomes more apparent when the reward time is shifted to a shorter duration (lower panel). Here, most responses are not initially rewarded and only the LTD related neuromodulator is released, triggered by the action.

Recently, a different protocol has been used for training rodents, a protocol that allows the researcher to estimate when the animals expect to receive a reward. In this protocol, a visual stimulus is delivered, the animals decide when to act (lick) after the visual cue, and the longer they wait the more reward (water) they get, up to a maximal reward time *t*_max_ after which they receive no reward (Figure 3A, red line) (Namboodiri et al., 2015). Animals trained with this protocol learn to delay their licking such that it is close to *t*_max_, and their chosen lick times are nearly optimal given the distribution of response times (Namboodiri et al., 2015). This protocol also results in changes to firing properties, similar to changes observed in previous protocols. Many of the cells in V1 exhibit a sustained increase in firing that terminates close to the time of action, and time of reward (Namboodiri et al., 2015).

We set out to examine if our two-trace learning rule is able to train a network with this training paradigm as well. To do this we used a similar network as in our previous simulations, and to this network we added a decision process that will trigger action. We used a very simple decision process such that once the average activity of excitatory cells in the network dips below a threshold (<15 Hz), action is initiated. We also assumed that reward is delayed by 200 ms, this delay is due to the time to initiate and execute the lick and also to the delay between the lick and the time that the reward signal arrives in the cortex (Hangya et al., 2015). While a delay is biologically realistic, some delay is also necessary for the simulations to succeed, and this delay must be larger than the crossing time between the LTP and LTD eligibility traces (see for example the crossing between the traces in Figure 2C, bottom).

With these assumptions, the network learns to increase synaptic efficacies and consequently increase the decay times of the neuronal responses. However, we find that this learning rule as it stands is insufficient to account for all the data. For instance, if the network is trained to one time interval such that the network initiates the action near the target time and then the reward time is changed to a shorter one, as illustrated by the upper and lower panels of Figure 3A, then the network will not be able to learn the new shorter reward time. This is because the action would be initiated at a time longer than the maximum reward time (lower panel in Figure 3A) and thus no reward would be given, and with the current learning rule the absence of reward results no change in synaptic efficacies. To address this we now consider, as exhibited experimentally (He et al., 2015), that there are two neuromodulators, one for expressing the LTP trace and the other for the LTD trace. We assume then that both of these neuromodulators are released when reward is delivered, however a much smaller release of the LTD associated neuromodulator is also assumed to be released upon action, even in the absence of reward.

Mathematically this means that if there is reward, we still use Equation (13) with $R^{p}\left(t\right)=R^{d}\left(t\right)=R\left(t\right)\delta \left(t-t_{\text{reward}}\right)$ at the time of reward. Where *R*(*t*) that defines the magnitude of reward has the form *R*(*t*) = *R*_*max*_(*t*/*t*_*max*_) for 0 < *t* < *t*_*max*_ and zero otherwise. However, if there is no reward, but action is initiated *R*^*p*^(*t*) = 0 but $R^{d}\left(t\right)=\kappa R_{max}\delta \left(t-t_{\text{action}}\right)$. The parameter κ << 1 scales the magnitude of neuromodulator in the absence of reward to be much smaller than that during reward, and this neuromodulator is delivered at the time the network initiates an action (*t*_action_). Figure 3B illustrates the neuromodulator release profile. During the reward interval, both neuromodulators are delivered (cyan for LTP and magenta for LTD) but only *R*^*d*^ during the action (the scale used here is intended for clarity and differs in the actual simulations). Although, the assumption that a moderate amount of LTD related neuromodulator is released in the absence of reward was chosen for mathematical convenience, it is consistent with experimental observations. The role of an LTD related neuromodulator could be played by serotonergic neurons in the dorsal raphe nucleus (DRN) which innervate the visual cortex (Koh et al., 1991). These neurons have been shown to respond differentially to the delivery or omission of reward (Li et al., 2013, 2016). Moreover, as shown in Li et al. (2016) DRN neurons ramp-up during the waiting time of delivery of reward, regardless of whether the reward is delivered, however they respond in a phasic way when the reward is delivered, but slowly decay to baseline activity.

**Figure 4:**
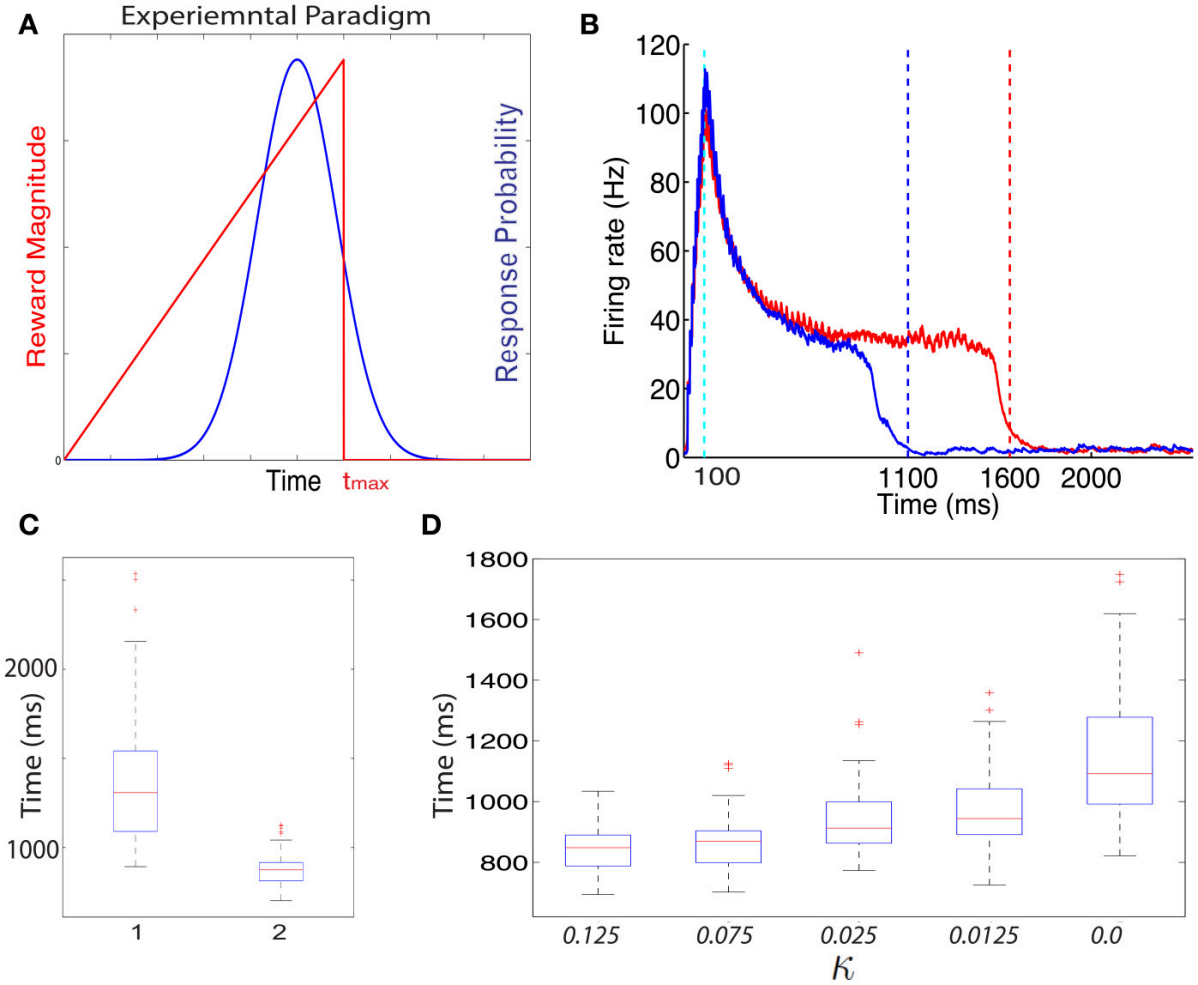
**Training with a ramp reward paradigm**. **(A)** In the ramp reward paradigm, the animal gets a reward that depends on the timing of its action. The magnitude of reward increases linearly with the timing of action, until a maximal time *t*_max_ after which no reward is obtained (red line). Rodents trained with this paradigm (Namboodiri et al., 2015) act close to the time *t*_max_ is a way that is nearly optimal given the temporal distribution of action times. A schematic depiction of the temporal distribution of action is shown by the blue curve. As observed experimentally, the peak of the response times is located slightly below *T*_max_. **(B)** Excitatory cells in a network trained with a ramp that terminates at *T*_max_ = 1500 ms exhibit sustained activity that terminates close to 1500 ms. A typical response is shown in red. When the paradigm is altered such that *t*_max_ = 1000 ms the neural response adapts and terminates close to 1000 ms (typical response in blue). **(C)** The neural response varies from trial to trial. A box-plot (median and quartiles) summarizes the statistics of threshold crossing for networks trained to 1500 ms (1) and 1000 ms (2). **(D)** The distribution of response times depends on the value of the parameter κ that determines the amount of *R*_2_ when no reward is delivered.

In Figure 4B we show the dynamics in a typical trial of the excitatory cells in this network after it is trained with the ramp-reward protocol with *t*_max_ = 1500 ms (red) (see Figure 4A). These dynamics can be altered when *t*_max_ is subsequently set to 1000 ms (Figure 4B, blue). Note that this reversal does not occur in a model in which only a single neuromodulator is used. The network dynamics change from trial to trial due to noise in the systems, consistent with experimental observations (Namboodiri et al., 2015). In Figure 4C we show summary statistics of the times that networks trained to 1500 ms (1) and 1000 ms (2) and cross the threshold from above. The medians obtained are 1308 ms for a network trained to 1500 and 875 ms for a network trained to 1000 ms. Note that we did not add the action delay to these numbers. These two distributions are statistically significantly different using an unpaired *t*-test, with *P* < 10^−10^.

The problem with the single neuromodulator model goes beyond its inability to transition back from long to short action times. In the absence of LTD expression, the response of a network trained from short to long action times will drift up. Eventually the network response time will move beyond *t*_max_. We can see this by simulating a network with a *t*_max_ = 1000 ms, setting κ = 0.0 and running the simulations for many more iterations. In such a case (see Figure 4D, right) the network median threshold crossing drifts up until it reaches ~1090 ms. The action will be delayed with respect to the neural response, which means that the action will be observed at longer that 1200 ms, and beyond *t*_max_. The slow drift to longer response times only ends when action times on every trial exceed *t*_max_.

Experimentally the distribution of action times peaks close to the optimal time to obtain maximal reward, given the width of the distribution of action times (Namboodiri et al., 2015). One could ask if a network trained with CRF and two distinct neuromodulator accomplishes this as well. We find that properties of the action time distribution, in our model, depend on the parameters of the model, most specifically on the value of κ. In Figure 4D we show how the properties of the distribution depend on the value of κ. As κ decreases, the location of the peak of the distribution increases. Therefore, our model does not obtain optimal performance for all parameters, but could approach optimality for a specific value of κ.

### 2.4. Two different neuromodulator signals are required to predict reward magnitude in a feed-forward network

**Figure 5:**
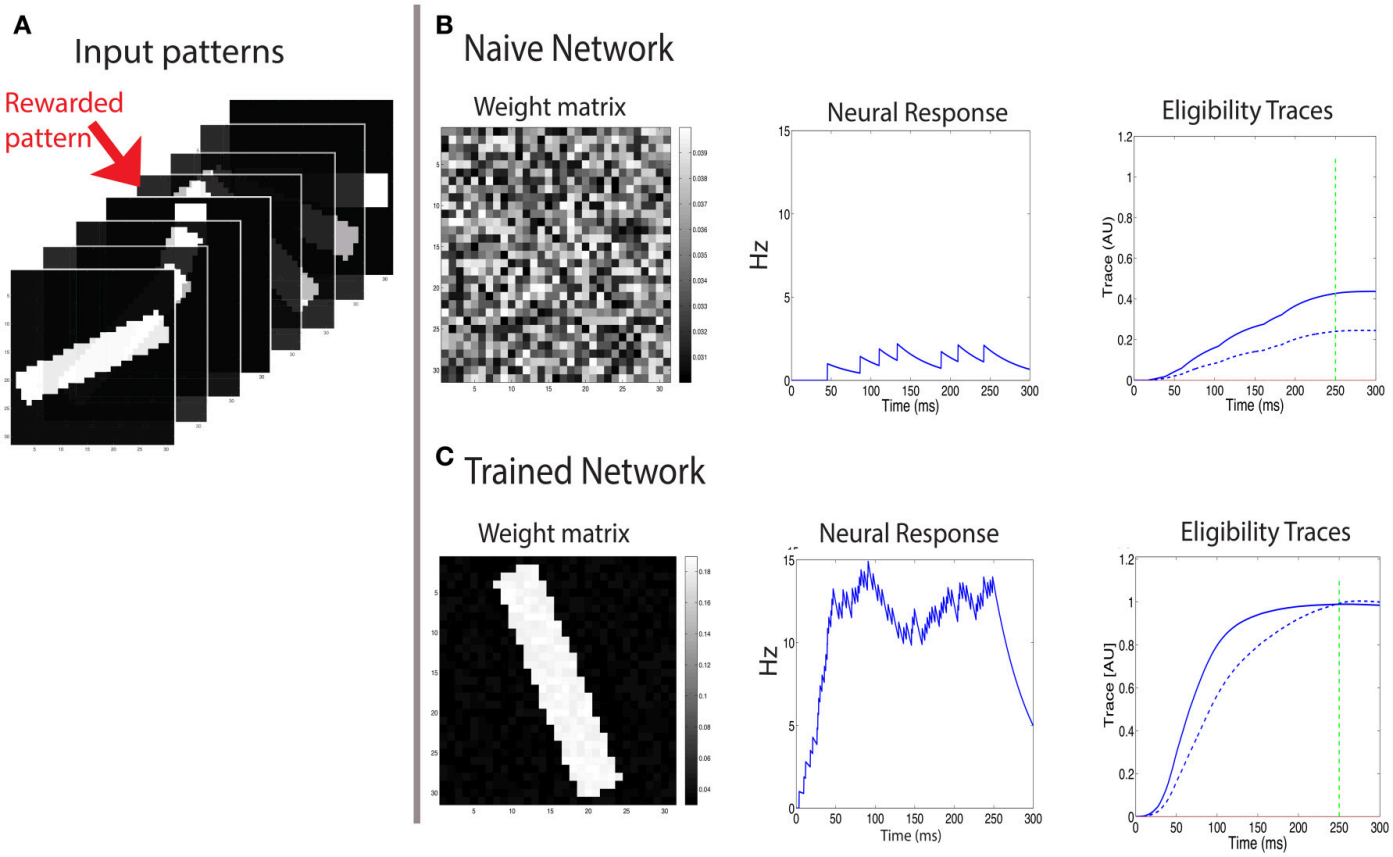
**Learning to respond to a rewarded pattern**. **(A)** A set of input patterns that are presented to the input layer, only one (red arrow) is rewarded. Other patterns are not rewarded. In this example, the number of patterns is *P* = 8, and the pattern dimension is 31 × 31. Here a discrete reward is delivered 250 ms after stimulus onset. **(B)** Before training, the weight vector is chosen to be random (left) and the response to all patterns is similar and weak (center). The two eligibility traces are increased due to the stimulus, and at the time of reward (green dashed line) the LTP trace (blue solid line) is stronger than the LTD trace (blue dashed line), resulting in potentiation of stimulated synapses. **(C)** After training, the weight vector has the same structure as the rewarded pattern (left), the response to the rewarded pattern (center) is strong, and the response to other patterns is much weaker. At the time of reward (vertical green dashed line), the LTP and LTD traces are equal in magnitude, such that no further changes in synaptic efficacies occur on average.

The recurrent network described above can code for the expected time of reward, but we have not shown that it can code for the magnitude of reward. To investigate this, we set up a one layer network that is exposed to various 2D input patterns (of size N x N) (Figure 5A). Only one of these inputs is rewarded. The reward can be delivered either continuously or discretely through the time during which the pattern is presented. We examine two simple cases, one in which the reward is delivered at one point in time, and another in which it has a constant value throughout the presentation of the stimulus.

At first we examine the consequences of training with a discrete reward signal for expressing the LTP and LTD traces of the form: $R^{p}\left(t\right)=R^{d}\left(t\right)=R\delta \left(t-t_{\text{reward}}\right)$. In this example the pattern is presented and remains on even after the time of the reward, which occurs at *t*_reward_ = 250 ms (see green line in Figures 5B,C). We find that this results in a potentiation of the weights that are stimulated by the rewarded pattern, while other weights are unchanged (Figure 5). Consequently the neuron becomes selective to the rewarded pattern. The strengthening of those synapses terminates once the LTP trace (*T*^*p*^) and the LTD trace (*T*^*d*^) are equal at the time of reward. Initially the weights vector is random (Figure 5B, left), the postsynaptic cell is only moderately activated (Figure 5B, center), and the LTP related trace *T*^*p*^ (solid line) is larger than the LTD trace *T*^*d*^ (dashed line) (Figure 5B, right), and both are much lower than saturation. These temporal profiles of the traces are determined by the model's parameters. For the *T*^*p*^ to increase faster than *T*^*d*^ we set η^*p*^ > η^*d*^. This faster increase of *T*^*p*^ is essential for the initial growth of weights stimulated by the rewarded pattern. Learning causes the weight vector to resemble the rewarded pattern (Figure 5C, left), and the postsynaptic activation due to that pattern is significantly increased (Figure 5C, center). Note that when learning stabilizes the two eligibility traces at the time of reward have the same value (Figure 5C, right) and this results in no further changes (on average) to synaptic efficacies.

It can be useful for the response magnitude to learn to represent the expected reward magnitude. One could assume that the magnitude of the neuromodulators depends on the reward magnitude. If both *R*^*p*^ and *R*^*d*^ depend on reward magnitude in the same way, the fixed point will be independent on the reward magnitude because the steady state depends on the ratio between *R*^*p*^ and *R*^*d*^. However, if they depend differently on reward magnitude then possibly reward magnitude could be represented in the response magnitude of the cells. We examine a simple case in which *R*^*p*^ depends on reward magnitude but the value of *R*^*d*^ is constant; it depends on the existence of reward, but not on the magnitude of reward.

**Figure 6:**
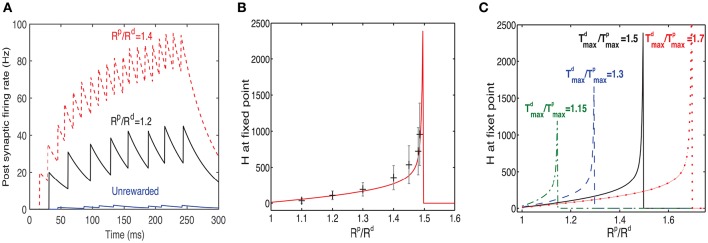
**Response to rewarded pattern can also learn to represent the magnitude of reward**. **(A)** Two examples for the postsynaptic neurons firing rate, in response to the rewarded pattern after training and one for the unrewarded case. In one example (black) *R*_*p*_/*R*_*d*_ = 1.2 and in the other (red) *R*_*p*_/*R*_*d*_ = 1.4. The unrewarded pattern (blue) has a much lower firing rate. This result illustrates that a network trained with two different ratios of LTP vs. LTD associated reward (*R*^*p*^/*R*^*d*^) learns to represent the specific ratios. When *R*^*p*^/*R*^*d*^ increases, so does the magnitude of the response to the rewarded pattern. Here $T_{\text{max}}^{d}/T_{\text{max}}^{p}=1.5$. **(B)** The value of *H* at steady state increases monotonically as a function of *R*^*d*^/*R*^*p*^ until a critical value is reached. Beyond this critical value the stable state is *H* = 0. The solid red line represents the analytical solution and the gray symbols represent the mean and standard deviation of simulations. The variability is over different synapses and time steps. All synapses associated with the rewarded pattern are taken into account. **(C)** *H* vs. *R*^*p*^/*R*^*d*^ (as in B) but for different values of $T_{\text{max}}^{d}/T_{\text{max}}^{p}$.

To examine the impact of changing the value of *R*^*p*^/*R*^*d*^, we ran several simulations in which we modified this ratio. The simulations were run until the system reached a fixed point, and no further significant changes in the receptive fields were observed. As the value of *R*^*p*^/*R*^*d*^ increased, so did the firing rate of the post-synaptic neuron in response to the rewarded pattern (see examples in Figure 6A), while the firing rates of non-rewarded patterns did not change significantly. On average the fixed point will occur when $R^{p}\left(t_{\text{reward}}\right)T_{ij}^{p}\left(t_{\text{reward}}\right)=R^{d}\left(t_{\text{reward}}\right)T_{ij}^{d}\left(t_{\text{reward}}\right)$. Since the reward occurs during the rising phase of the traces, the fixed point (see Equation 8) is reached when:

(17) $$\begin{array}{r} R^{p}{\tilde{T}}_{ij}^{p}\left(1-e^{-t_{\text{reward}}/{\tilde{\tau }}_{ij}^{p}}\right)=R^{d}{\tilde{T}}_{ij}^{d}\left(1-e^{-t_{\text{reward}}/{\tilde{\tau }}_{ij}^{d}}\right). \end{array}$$

Note that according to Equations (10, 11) ${\tilde{T}}^{p}$, ${\tilde{T}}^{d}$ as well as ${\tilde{\tau }}^{p}$ and ${\tilde{\tau }}^{d}$ depend on *H*. Therefore, solving Equation (17) is equivalent as solving for *H* at the fixed point. The Hebbian term, in its simplest form, is proportional to the activity of the output neuron, therefore knowing the value of *H* gives an indirect measure of the network's response. Consequently, by solving numerically for *H* at different levels of *R*^*p*^/*R*^*d*^ one can find how the magnitude of the Hebbian term (and thus the network response) at fixed point depends on the reward. A fixed point for this equation can be found if $R^{p}{\tilde{T}}_{ij}^{p}<R^{d}{\tilde{T}}_{ij}^{d}$ while at the same time ${\tilde{\tau }}^{p}<{\tilde{\tau }}^{d}$. Although, ${\tilde{T}}^{p}<T_{\text{max}}^{p}$ and ${\tilde{T}}^{d}<T_{\text{max}}^{d}$ (see Equation 10), they typically saturate close to these maximal values, due to the large value of the Hebbian term with respect to the saturation levels, i.e., $H\gg T_{\text{max}}^{\text{a}}$. This implies new approximately necessary conditions for a fixed point $R^{p}T_{\text{max}}^{p}<R^{d}T_{\text{max}}^{d}$. Note that the value of ${\tilde{\tau }}^{p}$ and ${\tilde{\tau }}^{d}$ depends on τ^*p*^ and τ^*d*^ respectively, but also on η^*p*^ and η^*d*^. By choosing appropriate values of η^*p*^ and η^*d*^ we can satisfy the condition ${\tilde{\tau }}^{p}<{\tilde{\tau }}^{d}$.

Although, Equation (17) cannot be solved in closed form, we can rewrite it in a simpler way by using the condition $H\gg T_{\text{max}}^{\text{a}}$ to give

(18) $$\begin{array}{l} \frac{r}{k}=\frac{1-e^{-\phi^{p}H}}{1-e^{-\phi^{d}H}} \end{array}$$

where *r* = *R*^*p*^/*R*^*d*^, $k=T_{\text{max}}^{d}/T_{\text{max}}^{p}$ and $\phi^{a}=t_{\text{reward}}/\left(\tau^{a}T_{\text{max}}^{a}\right)$. From this expression we can see that as *r* approaches *k* from below, then *H* → ∞, indicating that an increase in reward can be represented by the network as an increase in the network's response expressed in the Hebbian term. Solutions for *r* > *k* do not exist and this is indicated in Figure 6 by a sudden drop of the function to zero.

In Figure 6B we compare results of simulations to the solution of Equation (17), for $T_{\text{max}}^{d}/T_{\text{max}}^{p}=1.5$. We find a good agreement between the simulation results, averaged over many synapses, to the solution obtained by directly solving Equation (17) (solid line). We also find a large variability in each single trial between the value of *H* at different synapses (the error bars represent standard deviation over synapses), as well as between the value of *H* across trials within the same synapse. The variability primarily stems from the highly stochastic nature of presynaptic firing in each presynaptic neuron of each trial.

In Figure 6C we show the dependence of *H* on *R*^*p*^/*R*^*d*^ for different values of $T_{\text{max}}^{d}/T_{\text{max}}^{p}$. These results confirm the stability condition ($R^{d}T_{\text{max}}^{d}>R^{p}T_{\text{max}}^{p}$) derived above.

In Figure 6A we showed via simulations how the postsynaptic firing rate depends on the *R*^*p*^/*R*^*d*^ ratio, but our analysis in Equation (17) and in Figures 6B,C is for the Hebbian term *H*. Note that the fixed point for *H* is the same, independent of how *H* is actually calculated in simulations, as long as *H* is a form that is able to train the network and reach the fixed point. The details of the Hebbian term *H* do matter however for the post-synaptic rate. Given a specific functional form of *H* and a given presynaptic firing rate for the rewarded pattern, one could invert *H* to find the post-synaptic rate. For example, if *H* is simply *H* = *c* · *r*_pre_ · *r*_post_, where *r*_pre_ and *r*_post_ are the pre- and post-synaptic firing rates, respectively and *c* a proportionality constant. Given that we define the fixed point as: *H* = *H*^fp^ the postsynaptic rate will take the form: $r_{\text{post}}=H^{\text{fp}}/\left(c\cdot r_{\text{pre}}\right)$. Clearly for more complex Hebbian terms, for instance terms that depend on spike times, this inversion will be more complicated. However, the fixed point for *H* ≠ 0, will only be reached if the functional form of *H* chosen can reach this fixed point.

**Figure 7:**
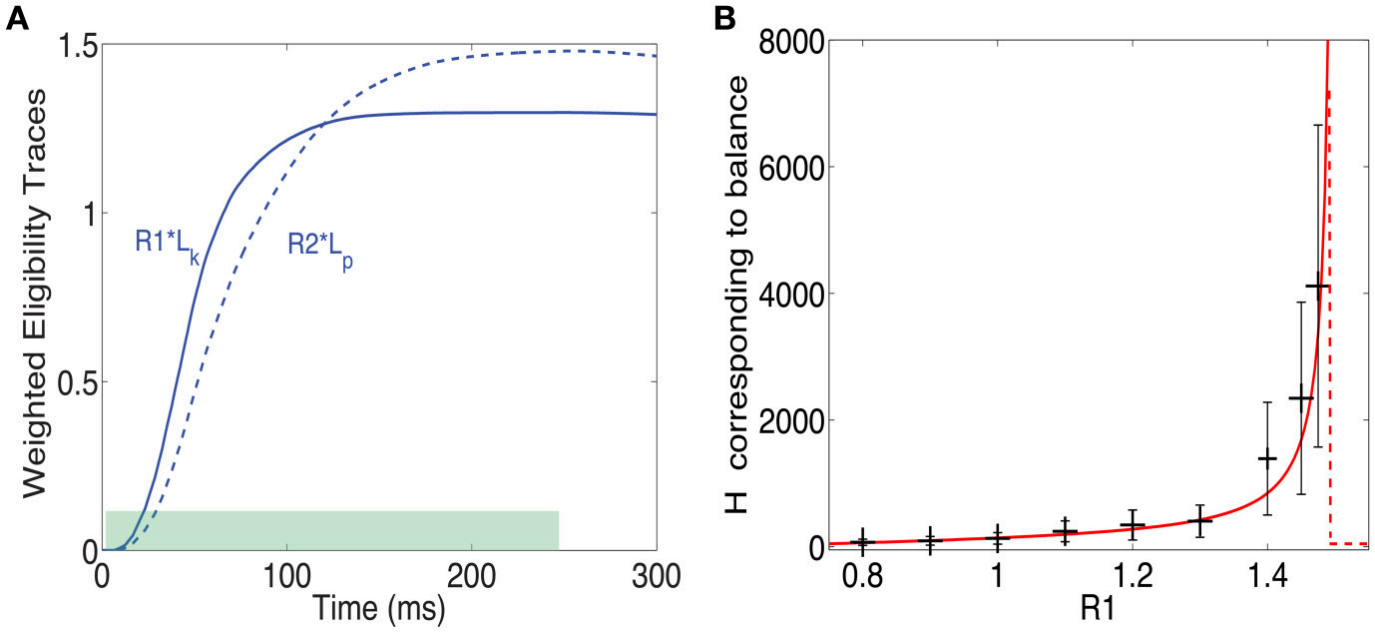
**Training with a step function reward**. **(A)** A pattern can be rewarded throughout the time it is presented (green shaded area), and not just at one time point as above. In such a case at steady state the integral of the LTP eligibility trace times its associated reward magnitude (solid line) is equal to the LTD eligibility trace times its associated reward (dashed line). **(B)** *H* at steady state vs. *R*^*p*^/*R*^*d*^ when $T_{\text{max}}^{d}/T_{\text{max}}^{p}=1.5$. The solid red line represents the analytical solution and the gray bars represent the mean and standard deviation of simulations. The variability is over different synapses and time steps.

The results until now assumed a reward delivered at one point in time (a δ function reward). This makes analysis simpler but is clearly unrealistic. Another simple option is that reward is delivered uniformly throughout and extended period between times *t*_1_ and *t*_2_ such that *t*_2_ − *t*_1_ = Δ*t*. These times represent the time interval during which reward is delivered, as illustrated in Figure 7A by the green bar at the bottom. Note that, as in the previous example, the reward is delivered while the pattern is still on. In that case the fixed point of Equation (13) takes the form:

(19) $$\begin{array}{r} R^{p}\int_{t_{1}}^{t_{2}}T_{ij}^{p}dt=R^{d}\int_{t_{1}}^{t_{2}}T_{ij}^{d}dt \end{array}$$

What this means is that the fixed point is attained when the integral of the LTP trace times its reward magnitude is equal to the integral of the LTD trace times the LTD reward magnitude (see Figure 7B). From these assumptions, one derives a fixed point equation of the form:

(20) $$\begin{array}{r} R^{p}{\tilde{T}}_{ij}^{p}\left(t_{2}-t_{1}+e^{-t_{2}/{\tilde{\tau }}_{ij}^{p}}-e^{-t_{1}/{\tilde{\tau }}_{ij}^{p}}\right)=\\ R^{d}{\tilde{T}}_{ij}^{d}\left(t_{2}-t_{1}+e^{-t_{2}/{\tilde{\tau }}_{ij}^{d}}-e^{-t_{1}/{\tilde{\tau }}_{ij}^{d}}\right). \end{array}$$

This equation, like in the δ function reward case, is implicitly a function of *H* and can be solved for *H*. We do not have a closed form solution for this equation, but it can easily be solved numerically. Note that to obtain this expression we made the approximation that *H* is constant throughout the integral. This approximation is worse here than for the δ function case and therefore one should expect less agreement between the simulations and the solution of Equation (20). In Figure 7B we compare the solutions of this equation to simulation results. There is a surprisingly good agreement between simulations and analysis despite the rough approximations. Not surprisingly, the agreement is worse than for the δ function reward shown above. Importantly, and irrespective of the precise details, we find that it is possible to learn the magnitude of reward with two different neural modulators and this is possible for different temporal profiles of the reward signal.

## 3. Discussion

We previously presented a theory (CRL) for stable reinforcement learning based on temporal competition between synaptic eligibility traces (He et al., 2015). According to this theory, learning occurs only in the presence of a neuromodulator that converts the activity level of these traces into changes in synaptic efficacy. Within our original framework, a single neuromodulator can result in either potentiation (LTP) or depression (LTD), depending on the magnitude of the different eligibility traces. When this model is embedded as the learning rule in a recurrent network, this theory is able to account for the formation of sustained cortical dynamics that can be used to predict reward timing. The key mechanism underlying this is the simultaneous expression and competition between potentiation and depression fostered by the presence of the rewarding signal (neuromodulator), which results in a net potentiation that eventually is fully balanced by the depression component. Stability of learning in our theory requires the coexistence of LTP and LTD related traces in single synapses. This assumption does not yet have explicit experimental support, since in the experiments reported in He et al. (2015) pure pre-post or post-pre induction protocols have been used. We expect that *in vivo* firing patters will be similar to those in our simulations, and will result in a mix of pre-post and post-pre coincidences, resulting in the coexistence of LTP and LTD related traces in single synapses.

While the theory as described requires a single neuromodulator, the existence of several neuromodulators has been observed experimentally (He et al., 2015). The question we address here is what purpose could multiple neuromodulators serve? First, we showed that a recurrent network, training via CRL with only one neuromodulator fails to account for results of a novel ramp-reward training paradigm (Namboodiri et al., 2015). The single neuromodulator CRL fails in learning to predict a short reward time after it previously learned a longer interval and it drifts when learning any reward time such that the network learns to predict reward times that fall after the end of the reward window. We expand the CRL model by adding separate neuromodulators for LTP and LTD traces, and by postulating that an LTD specific neuromodulator can be active upon action in the absence of reward. This revised CRL model is able to learn the appropriate reward times and to learn short reward times after a previous long reward was learned. There is significant scientific evidence regarding the temporal responses of dopamine neurons (Schultz et al., 1997) although recent results are further expanding our knowledge (Howe and Dombeck, 2016). Not as much is known about the temporal dynamics of other neuromodulators in response to reward, although recent evidence has emerged that ACh releasing neurons in the basal forebrain have a rapid response to negative and positive rewards (Hangya et al., 2015). We hypothesize that some neuromodulators that are necessary for reward dependent LTD could be released upon action and in the absence of reward; such a prediction should be experimentally tested.

In a second example we set out to examine if CRL can learn other parameters of a predictable reward, for example the magnitude of reward. For simplicity and in order to make a more general statement we tested this in a feedforward network that could conceptually predict the magnitude of reward, but not its expected timing. We first showed that a single neuromodulator CRL is able to train such a network to respond selectively to the rewarded input pattern, and that such learning is stable. However, it is unable to represent the magnitude of reward, because the fixed point is independent of this parameter. In contrast, a modified CRL with two neuromodulators that respond differentially to the reward can learn to represent reward magnitude. We also characterized the parameter ranges that enable such learning. We have previously shown how a network of of spiking excitatory and inhibitory neurons, trained with our previous learning rule, develops all the different cell types observed in the visual cortex experiments (Huertas et al., 2015). This network can also develop one class of cells, cells with a peaked response at the time of reward, that can also predict reward magnitude. The learning rule used in that model depended on the inhibition of the reward signal. Further work should examine if the expanded CRL proposed here, when embedded in a similar recurrent network, can also result in the different types of cells, as observed experimentally (Shuler and Bear, 2006; Chubykin et al., 2013; Namboodiri et al., 2015).

## 4. Methods

We will describe here both the recurrent and the feedforward networks. All neurons are leaky integrate and fire neurons, whose membrane potential is governed by the equations:

(21) $$\begin{array}{r} C\frac{d\nu_{i}^{\text{p}}}{dt}=g_{\text{L}}\left(E_{\text{L}}-\nu_{i}^{\text{p}}\right)+g_{\text{E},\text{i}}\left(E_{\text{E}}-\nu_{i}^{\text{p}}\right)+g_{\text{I},\text{i}}\left(E_{\text{I}}-\nu_{i}^{\text{p}}\right) \end{array}$$

(22) $$\begin{array}{r} \frac{ds_{k}}{dt}=-\frac{1}{\tau_{\text{s}}}s_{k}+\rho \left(1-s_{k}\right)\underset{\text{l}}{\sum }\delta \left(t-t_{\text{l}}^{k}\right) \end{array}$$

where $\nu_{i}^{\text{p}}$ represents the membrane potential of the *i*-th neuron in population *p*, which can be either excitatory (E) or inhibitory (I), and where *s*_*k*_ is the synaptic activation of the *k*-th pre-synaptic neuron. Other parameters are: membrane capacitance (*C*); leak, excitatory, and inhibitory conductances (*g*_L_, *g*_E, i_, *g*_I, i_); leak, excitatory, and inhibitory reversal potentials (*E*_L_, *E*_E_, *E*_I_); percentage change of synaptic activation with input spikes (ρ) and time constant for synaptic activation (τ_s_). Different synaptic time constants were used in different neurons types and in the different models. The delta-function in Equation (22) indicates that these changes occur only at the moment of the arrival of a pre-synaptic spike at $t_{\text{l}}^{k}$ where the index *l* says that this is the *l*'th spike in neuron *k*.

The total excitatory and inhibitory conductances are computed from the individual outgoing synaptic activations, *s*_*k*_, and the synaptic strength, Ω_*ik*_, between the post-synaptic neuron *i* and the pre-synaptic neuron *k*. Thus, for both excitatory and inhibitory currents we have

(23) $$\begin{array}{r} g_{\text{E},\text{i}}=\underset{k}{\sum }\Omega_{ik}^{\text{E}}s_{k} \end{array}$$

(24) $$\begin{array}{r} g_{\text{I},\text{i}}=\underset{k}{\sum }\Omega_{ik}^{\text{I}}s_{k} \end{array}$$

where the index *k* runs over all pre-synaptic neurons (either from the excitatory or inhibitory populations accordingly) contacting the post-synaptic neuron *i* which for *g*_E,i_ is either an excitatory or an inhibitory neuron and for *g*_I,i_ is an excitatory neuron.

In the simplest recurrent network (Figure 2A) we have only excitatory neurons, and the time constant we use is as in our previous work (Gavornik et al., 2009) τ_s_ = 80 ms. The excitatory feedforward synapses have a time constant of τ_s_ = 10 ms. In the network with both excitatory and inhibitory connections (Figure 2B), the time constant of inhibitory synapses is τ_s_ = 10 ms. More details about this network can be found in Huertas et al. (2015).

In the feedforward network there is only a single postsynaptic integrate and fire neuron with τ_s_ = 10 ms. In all cases input patterns set the rate for a Poisson process that generates random spikes in the afferent feedforward synapses. To calculate the Hebbian term that generates the traces, we first compute an estimate of the rate in each neuron using the following process:

(25) $$\begin{array}{r} \tau_{r}\frac{dr_{i}\left(t\right)}{dt}=-r_{i}\left(t\right)+S_{i}\left(t\right) \end{array}$$

where *S*_*i*_(*t*) represents the spike times in neuron *i* and has the form $S_{i}\left(t\right)=\underset{k}{\sum }\delta \left(t-t_{k}^{i}\right)$, where $t_{k}^{i}$ is the timing of spike number *k* in neuron *i*. Here we used τ_*r*_ = 50*ms*, results are insensitive to the precise choice.

A copy of the code used to perform the simulations in the paper can be requested from the authors.

## Author contributions

MH and HS conceived the project; MH, SS, and HS wrote code, ran simulations, carried out the analysis and wrote the paper.

## Funding

This work was funded in part by ONR grant N00014-16-R-BA01.

### Conflict of interest statement

The authors declare that the research was conducted in the absence of any commercial or financial relationships that could be construed as a potential conflict of interest.

## References

[B1] Beitel R. E. Schreiner C. E. Cheung S. W. Wang X. Merzenich M. M. (2003). Reward-dependent plasticity in the primary auditory cortex of adult monkeys trained to discriminate temporally modulated signals. Proc. Natl. Acad. Sci. U.S.A. 100, 11070–11075. 10.1073/pnas.1334187100 12941865 PMC196928

[B2] Cassenaer S. Laurent G. (2012). Conditional modulation of spike-timing-dependent plasticity for olfactory learning. Nature 482, 47–52. 10.1038/nature10776 22278062

[B3] Chubykin A. A. Roach E. B. Bear M. F. Shuler M. G. H. (2013). A cholinergic mechanism for reward timing within primary visual cortex. Neuron 77, 723–735. 10.1016/j.neuron.2012.12.039 23439124 PMC3597441

[B4] Gavornik J. P. Shouval H. Z. (2010). A network of spiking neurons that can represent interval timing: mean field analysis. J. Comput. Neurosc. 30, 501–513. 10.1007/s10827-010-0275-y 20830512 PMC3059329

[B5] Gavornik J. P. Shuler M. G. H. Loewenstein Y. Bear M. F. Shouval H. Z. (2009). Learning reward timing in cortex through reward dependent expression of synaptic plasticity. Proc. Natl. Acad. Sci. U.S.A. 106, 6826–6831. 10.1073/pnas.0901835106 19346478 PMC2672535

[B6] Hangya B. Ranade S. P. Lorenc M. Kepecs A. (2015). Central cholinergic neurons are rapidly recruited by reinforcement feedback. Cell 162, 1155–1168. 10.1016/j.cell.2015.07.057 26317475 PMC4833212

[B7] He K. Huertas M. Hong S. Z. Tie X. Hell J. W. Shouval H.. (2015). Distinct eligibility traces for LTP and LTD in cortical synapses. Neuron 88, 528–538. 10.1016/j.neuron.2015.09.037 26593091 PMC4660261

[B8] Howe M. W. Dombeck D. A. (2016). Rapid signalling in distinct dopaminergic axons during locomotion and reward. Nature 535, 505–510. 10.1038/nature18942 27398617 PMC4970879

[B9] Huertas M. A. Hussain Shuler M. G. Shouval H. Z. (2015). A simple network architecture accounts for diverse reward time responses in primary visual cortex. J. Neurosci. 35, 12659–12672. 10.1523/JNEUROSCI.0871-15.2015 26377457 PMC4571602

[B10] Koh T. Nakazawa M. Kani K. Maeda T. (1991). Association with reward negatively modulates short latency phasic conditioned responses of dorsal raphe nucleus neurons in freely moving rats. Brain Res. Bull. 27:675. 23486976 10.1523/JNEUROSCI.5679-12.2013 PMC6618993

[B11] Li Y. Dalphin N. Hyland B. I. (2013). Association with reward negatively modulates short latency phasic conditioned responses of dorsal raphe nucleus neurons in freely moving rats. J. Neurosci. 11, 5065 10.1523/JNEUROSCI.5679-12.2013 PMC6618993 23486976

[B12] Li Y. Zhong W. Wang D. Feng Q. Liu Z. Zhou J.. (2016). Serotonin neurons in the dorsal raphe nucleus encode reward signals. Nat. Commun. 7:10503. 10.1038/ncomms10503 26818705 PMC4738365

[B13] Liu C.-H. Coleman J. E. Davoudi H. Zhang K. Hussain Shuler M. G. (2015). Selective activation of a putative reinforcement signal conditions cued interval timing in primary visual cortex. Curr. Biol. 25, 1551–1561. 10.1016/j.cub.2015.04.028 26004763 PMC4470722

[B14] Namboodiri V. M. K. Huertas M. A. Monk K. J. Shouval H. Z. Hussain Shuler M. G. (2015). Visually cued action timing in the primary visual cortex. Neuron 86, 319–330. 10.1016/j.neuron.2015.02.043 25819611 PMC4393368

[B15] Schultz W. Dayan P. Montague P. R. (1997). A neural substrate of prediction and reward. Science 275, 1593–1599. 9054347 10.1126/science.275.5306.1593

[B16] Shuler M. G. Bear M. F. (2006). Reward timing in the primary visual cortex. Science 311, 1606–1609. 10.1126/science.1123513 16543459

[B17] Sutton R. S. Barto A. G. (1998). Reinforcement Learning: An Introduction. Cambridge, MA: MIT Press.

[B18] Yagishita S. Hayashi-Takagi A. Ellis-Davies G. C. R. Urakubo H. Ishii S. Kasai H. (2014). A critical time window for dopamine actions on the structural plasticity of dendritic spines. Science 345, 1616–1620. 10.1126/science.1255514 25258080 PMC4225776

[B19] Yin P. Fritz J. B. Shamma S. A. (2014). Rapid spectrotemporal plasticity in primary auditory cortex during behavior. J. Neurosci. 34, 4396–4408. 10.1523/JNEUROSCI.2799-13.2014 24647959 PMC3960477

